# Linguistic explanation and domain specialization: a case study in bound variable anaphora

**DOI:** 10.3389/fpsyg.2015.01421

**Published:** 2015-09-24

**Authors:** David Adger, Peter Svenonius

**Affiliations:** ^1^Linguistics, Queen Mary University of LondonLondon, UK; ^2^Center for Advanced Study in Theoretical Linguistics, Department of Language and Linguistics, University of Tromsø – The Arctic University of NorwayTromsø, Norway

**Keywords:** universal grammar, domain specificity, bound variable anaphora, syntax semantics interface

## Abstract

The core question behind this Frontiers research topic is whether explaining linguistic phenomena requires appeal to properties of human cognition that are specialized to language. We argue here that investigating this issue requires taking linguistic research results seriously, and evaluating these for domain-specificity. We present a particular empirical phenomenon, bound variable interpretations of pronouns dependent on a quantifier phrase, and argue for a particular theory of this empirical domain that is couched at a level of theoretical depth which allows its principles to be evaluated for domain-specialization. We argue that the relevant principles are specialized when they apply in the domain of language, even if analogs of them are plausibly at work elsewhere in cognition or the natural world more generally. So certain principles may be specialized to language, though not, ultimately, unique to it. Such specialization is underpinned by ultimately biological factors, hence part of UG.

## 1. Introduction

A core question in the cognitive science of language is whether explaining linguistic phenomena requires appeal to properties of human cognition that are specific to the language-using capacity of human beings. A common approach is to propose that domain general principles are at play in language without showing how these principles have the empirical reach of well established generalizations known within linguistics (Bybee and McClelland, 2005; Christiansen and Chater, 2015). This is not a strategy that is likely to lead to progress. A more promising alternative is to attempt to match up known generalizations about language with proposals about domain general principles (e.g., Culicover and Jackendoff, 2012). It seems to us, however, that a reasonable way to answer the question of domain specificity, given the current state of knowledge in cognitive science, is to develop theoretical approaches to linguistic phenomena which have as much empirical reach and explanatory depth as possible, and to evaluate the posits of such theories for domain generality. That third approach is what we engage in here.

There is nothing particularly totemic in the issue, at least from the perspective of generative syntax. We should hope that aspects of our best theories of syntactic phenomena are simply special cases of more general principles. But those more general principles are not established at the moment, at least not in such a way as provide deep explanations of even rather elementary properties of human syntax. Indeed, we think that generative syntax provides a potential way to reach those more general principles, and that human language is a particularly rich domain for the development of theories of some depth that may allow us to glimpse any deeper underlying regularities. The goal of this article is, then, to present a well-developed theoretical proposal for an important linguistic phenomenon and to show how the principles that underpin the proposal reveal that abstract, high-level principles of the computational construction of pairings of sound and meaning are at play. We then evaluate whether these principles are specific to language, concluding that the principle that licenses linguistic structures is plausibly so, while the principles that regulate how structures are interpreted are at least specialized to language, though they may be not even specific to cognition.

We will make the general argument here through the phenomenon of bound variable anaphora. The argument goes as follows: (i) the phenomenon is a real phenomenon of human language in general; (ii) there is a compelling generative theory that limns its empirical contours rather exactly; (iii) there are no equally empirically wide or theoretically compelling competing accounts; (iv) some explanatory devices in the successful theory appear to be specialized for language, as far as current understanding goes (even if analogs of them may be observed elsewhere in cognition).

Often generative syntactic analyses can be impenetrable to those trained outside of the discipline, so we attempt here to drill down to the core essentials and to make these accessible, drawing out the more general theoretical implications for cognition, and examining to what extent the theoretical principles we use are specific to linguistic cognition.

## 2. Structural constraints on interpretation

### 2.1. Introducing bound variable interpretations

The phenomenon we will use to make the argument here is known as bound variable anaphora. Take the English sentence in (1):

(1) No woman denies that she has written a best selling novel.

What is the meaning of this sentence? There are two that are readily discernible (Evans, 1980). One is that, from a group of women, not one denied that some individual (say Julie) had written a best selling novel. This meaning is easily accessible given either a preceding discourse to provide context, or, an individual that is salient in the context where the sentence is uttered. For example:

(2) Hello everyone. This is Julie, who's recently been in the news again. Now, no woman denies that she has written a best selling series of novels featuring female protagonists, but some deny that these novels are good for equal rights.

Following Evans, we'll call this meaning, where the pronoun receives its interpretation from the context, the *referential* meaning.

The second meaning is simply that, if you have a group of women, and you check all of them one by one, you will not find any who deny that they themselves have written a best selling novel. This is called the *bound variable* meaning.

We also find this ambiguity effect with quantifier phrases containing quantifiers other than *no*. For example, all of the following sentences have the same ambiguity; the pronoun can have a referential or a bound variable interpretation:

(3)
  - Every woman said she had met the Shah.
  - Did any woman say that she had met the Shah?
  - Every woman persuaded her son to organize her birthday party.
  - Each author decided that she should be at the signing.

We find bound variable anaphora in various languages (Déchaine and Wiltschko, 2014). For example, the Algonquian language Passamaquoddy displays the same effect (Bruening, 2001):

(4) Psi=te     wen        litahasu eli   w-itapi
       all=emph someone think.3  that 3-friend.obvp
       woli-pomawsuwin-uw-ulti-htit
       good-person-be-plural-3pconj
       “Everyone thinks his friends are good people.”
(5) Ma=te       wen        litahasi-w     nekom mahtoqehs.
       neg=emph someone think.3-neg he        rabbit
       “No one thinks he's a rabbit.”
(6) Ma=te       wen        ʔ-kosiciy-a-wiy-il            eli
       neg=emph someone 3-know.ta-dir-neg-obv that
       Maliw-ol muhsal-iht.
       Mary-obv like-3conjinv
       “No one knows that Mary likes him.”

The following examples from Scottish Gaelic also show the same effect:

(7) Thuirt       gach caileag gu   robh       i    a'
        say.past each girl       that be.past she prog
        faireachdainn   tinn.
        feeling             sick
        “Every girl said she was feeling sick.”
(8) Cha robh       caileag sam bith   ag      ràdh gu   robh
        neg be.past girl       in    being prog say   that be.past
        i     tinn.
        she sick.
        “No girl said she was sick.”

We have given these non-English examples to show that this phenomenon is not simply a grammatical quirk of English or other well studied European languages. The exact empirical contours of bound variable anaphora, as outlined here and explained below, are not, however, detectable in every language. For a language to display this particular pattern, it needs to have determiner quantifiers, which not all languages possess (Bach et al., 1995). Further, it must have a determiner quantifier that is singular. English has both singular determiner quantifiers (as in “every boy”) and plural ones (e.g., “all boys”). Some languages, however, lack singular determiner quantifiers. Further, the language must ideally be able to use singular pronouns with the singular quantifier to create the relevant reading. This is also not available to all languages. Indeed, in English, the plural pronoun is often used in informal discourse, especially when the gender of the quantified noun phrase is unknown or avoided: for example “Every author was able to choose their own cover.” In such circumstances, the plural pronoun can be construed as referring to a group of individuals that is constructed out of all the authors, similarly to the behavior of *they* in following discourse in English: “Every author was grumpy. They had been locked out of the decision about their book covers” (Kamp and Reyle, 1993; Rullmann, 2003). The existence of this strategy makes discerning true bound variable readings with plural pronouns challenging. Beyond these basic requirements, languages place various other restrictions on their pronouns which mean that quite careful investigation is required to determine whether there is a bound variable construction. However, we can control for these relevant factors by cross-linguistic investigation, and when the various conditions listed are met, the phenomenon reveals itself to be very consistent.

Bound variable interpretations of pronouns, then, arise when the meaning of a singular pronoun is dependent in a particular way on the meaning of a singular quantifier phrase elsewhere in the sentence (the importance of number and person features for bound variable meanings across languages is discussed in Kratzer (2009), Adger (2011); see Harbour (2014) for a compatible theory of grammatical number). When a bound variable interpretation is available in the examples we have seen, a referential interpretation is also available, leading to the ambiguity.

Let us turn now to structural constraints on the availability of this interpretation. In certain cases, it turns out that the bound variable meaning vanishes, and only the referential reading is left. For example:

(9)
  - A man who no woman likes denies that she has written a best selling novel.
  - The man that every woman loved said she had met the Shah.
  - The man that didn't love any woman said she had met the Shah.
  - That every woman seemed so sad persuaded me to organize her birthday party.
  - Because every author hates you, she will try to kill you.

If one pauses to think about the meanings of these sentences, it turns out that they are not interpreted as involving the pronoun's meaning varying with the quantifier in the way we have just seen. Compare, for example, (9-c) with (3-a). (3-a) can be paraphrased as “Given a set of women salient in the context, for each choice of some woman you make from that set, that woman you have chosen said that she herself had met the Shah.” A corresponding paraphrase for (9-c) would be “Given a salient set of women in the context, for each choice you make from that set, the man that didn't love the woman you have chosen said that that that woman had met the Shah.” But that paraphrase doesn't capture the meaning of the sentence in (9-c). In fact, the sentence only has a paraphrase that goes something like “Given a salient set of women in the context, the man that didn't love any woman you may choose from that set said that that *she*—some other female person in the context—had met the Shah.” That is, the pronoun *she* is not ambiguous between the two interpretations: it is only referential. This is an odd meaning out of context, but is the only meaning available.

This same effect holds for the other sentences, and countless more pairs like them. Although we have illustrated the phenomenon just by appealing to what meanings are intuitively available for sentences here, it is experimentally robust (Kush et al., 2015).

We also see bound variable readings disappear in Passamaquoddy and in Scottish Gaelic, in certain circumstances. (The ^*^ in the examples here marks not ungrammaticality, but rather the unavailability of the bound variable reading).

(10) ^*^Ipocol   psi=te      wen        Sipayik k-nacitaham-oq,
           because all=emph someone Sipayik 2-hate-inv
           kt-oqeci=hc nehpuh-uk
           2-try=fut    kill-inv
           “Because everyone at Sipayik hates you, he will try to kill you.”

And in Gaelic

(11)
  ^*^Thuirt      duine a    bhruidhinn ris gach caileag
      say.past man  that spoke       to each girl
      gun robh       i    tinn
      that be.past she sick
      “A man that was talking to each girl said she was sick.”
  ^*^Air sgath 's gun do bhuail thu gach balach,
      because     that hit.past  you each boy
      ruith       e   air falbh
      run.past he away
      “Because you hit each boy, he ran away.”

In examples like those in (9), (10), and (11), the quantifier precedes the pronoun just as it does in the examples in (1) and (3). However, the bound variable reading is available in (1) and (3) and is unavailable in (9), (10), and (11). So the issue is not (merely) one of precedence. Various proposals have been put forward in the generative literature as to what, exactly, is responsible for the difference. The current consensus is that there are two interrelated factors involved: semantic scope and syntactic command (Safir, 2004; Barker, 2012; Déchaine and Wiltschko, 2014).

### 2.2. Scope

Scope is simply a name for the fact that the interpretation of certain units of language is computed as a subpart of the interpretation of larger units, a cognitive factor that plausibly exists elsewhere than in language. The larger unit is said to take wide scope over the smaller unit. Consider the following cases:

(12)
  - An author read every book.
  - An author thought every book was good.
  - An author thought Julie had read every book.

In (12-a), there are two meanings. In one meaning, we interpret the phrase *an author* as dependent on the interpretation we provide for *every book*; that is, the semantic computation that builds the meaning of *every book* includes a meaning assigned to *an author*. In the other, the dependency is the other way around. We can make this intuition explicit by sketching a procedure to compute the meaning of the quantifier phrases. Let us take a simpler example first:

(13) Every book is interesting.

We can treat computing the meaning of *every book* as involving three separate computational procedures (Peters and Westerståhl, 2006):

(14)
  - Identify a salient set in the context of the discourse; in this case a set of books (this set is called the “restriction” of the quantifier).
  - Identify the property which is characterized by the “scope” (the rest of the clause)—in this case, being interesting.
  - Apply a quantificational operator (in this case *every*) to determine whether every element of the set of books is such that the property of being interesting holds of it.

Similarly, we compute the meaning of *an author* by taking a set of authors and checking whether a condition represented by the rest of the sentence holds of one of the elements of that set.

(15) An author won this week's lottery.
(16)
  - Identify a salient set in the context of the discourse; in this case a set of authors.
  - Identify the property which is characterized by the “scope” (the rest of the clause)—in this case, winning this week's lottery.
  - Apply a quantificational operator (in this case *an*) to determine whether at least one element of the set of authors is such that the property of winning this week's lottery holds of that element.

These trivial cases are then put together for our example (12-a). We can take either the set of books first, and then compute the condition that holds of every book as involving an author, or we can take an author first, and then see whether the condition involving every book holds of an author. This gives us two distinct meanings.

Let's take *every book* first:

(17) Take a set of books salient in the context. Now go through the books one by one, and for each choice you make of a book, see whether an author (from a salient set of authors) has read that book. Going through the set of books, ensure that for all of the choices of book some author has read the book chosen.

This process implies that it is possible to have a different author for each book. This is the *wide scope* reading for *every*, as the computation of *an author* takes place within the computation for *every book*. The other meaning of *an author read every book* works out as follows:

(18) Take a set of authors salient in the context. Now go through the authors one by one and for each choice made, go through the set of books salient in the context and see whether the author you have chosen has read every member of the set of books. Ensure that there is at least one author of whom this condition holds.

This is the *narrow scope* reading for *every*. The crucial empirical difference is that in the wide scope reading for *every book*, we can have a different author picked for each different book, while in the narrow scope reading, once we've picked our author, that author needs to have read every book for the interpretation to be true.

It turns out that there are structural constraints on the scope of quantifiers. Consider the sentence in (12-b): this doesn't have the wide scope reading for *every*. Neither does the sentence in (12-c). This is because a quantifier cannot scope outside the tensed clause it is in. This idea, that certain semantic effects are bound into local syntactic domains, is of venerable descent in linguistics, originally due to Langacker (1969). We'll call it the Command Generalization:

(19) The Command Generalization: A quantifier scopes over everything in the minimal finite clause it appears in.

### 2.3. Applying scope to bound variables

The generalization that seems to be most effective in determining when a quantifier phrase can bind a pronoun is the following (this is just a descriptive generalization, not a theory, as yet):

(20) The Scope Generalization: For a quantifier to bind a pronoun it must scope over that pronoun.

For example, consider the following example:

(21) Every woman says that she has written a best selling novel.

This sentence has the following rough paraphrase: take a set of women. Now go through that set one by one, and see whether, for each choice of a woman, that woman said that she, herself, wrote a best selling novel. For the sentence to come out true, all of the choices of individuals from the set of women should work.

Now compare that to the following case:

(22) A man who every woman likes says that she has written a best selling novel.

If the quantifier phrase *every woman* could scope over the rest of the sentence, it should be able to bind the pronoun. But we can independently tell that *every woman* is restricted in its scope. If we put a quantifier phrase like *an author* in place of *she*, we get:

(23) A man who every woman likes says that an author has written a best selling novel.

We can see that *every woman* doesn't, descriptively, scope over *an author*, because the sentence doesn't have a reading where the authors potentially change for each choice made from the set of women. So the Scope Generalization correctly correlates the capacity of a quantifier to scope over the pronoun with its ability to bind the pronoun. The Command Generalization captures why the quantifier doesn't have wide scope over the pronoun in this sentence: the quantifier is “trapped” within the finite (relative) clause *who every woman likes*.

Together, the Scope Generalization and the Command Generalization do a good job of capturing the data we have seen. Consider again, our first example:

(24) No woman denies that she has written a best selling novel.

Here, the smallest finite clause containing the quantifier phrase *no woman* is the whole sentence. That sentence contains a further clause *that she has written a best selling novel* and that clause contains the pronoun. So *no woman* scopes over the pronoun *she* and *she* can therefore have a bound reading, in the way described above. For the sake of visualization, we can represent this as a tree-like structure, where the scope of a quantifier phrase is its sister in the tree:

(25)
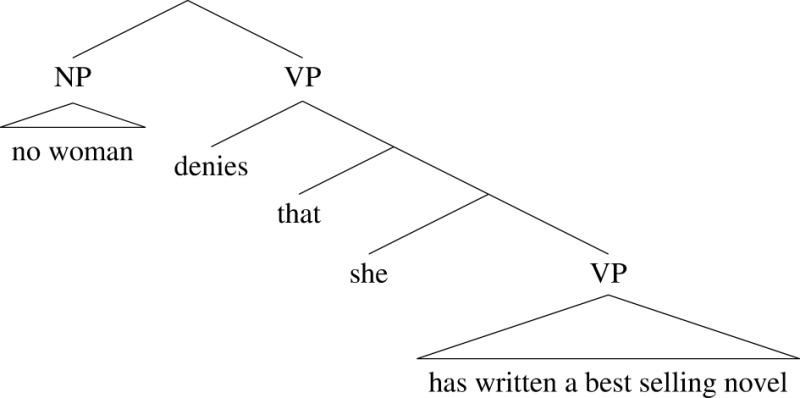

Compare this with the corresponding example from (9), which lacks a bound variable interpretation:

(26) A man who no woman likes denies that she has written a best selling novel.

*No woman* is in a finite (relative) clause of its own *who no woman likes*. It cannot therefore take scope over the whole sentence, so the pronoun *she* cannot be bound. Again, we can visualize the structure in a tree-like fashion:

(27)
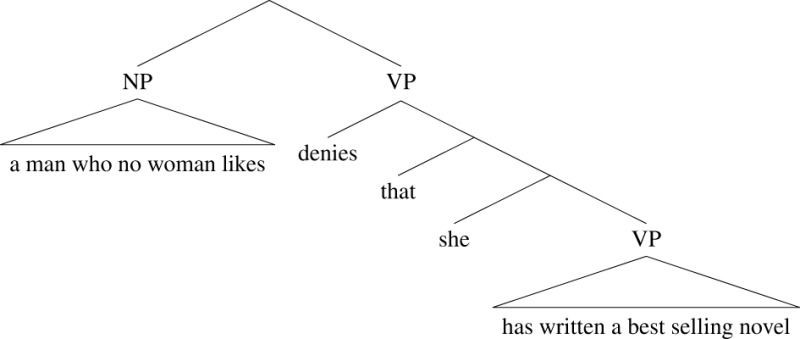

Here the scope of the quantifier phrase is again its sister in the tree, but the sister of *no woman* is just the verb *likes*, and so the quantifier phrase does not scope over the pronoun.

Our descriptive generalizations also capture the fact that the bound reading vanishes in examples like the following:

(28)
  - She persuaded the Shah that every woman should be imprisoned.
  - She didn't believe that I had been introduced to any woman.
  - She expected that each author's book signing would be private.

Here, the quantifier phrases are inside an embedded finite clause, and the Command Generalization stops them scoping over the whole sentence, so the pronoun cannot be bound. (28-a), for example, can't have a paraphrase where for each individual chosen from a set of women, that individual persuaded the Shah to imprison her.^1^

Summarizing, we have seen that the phenomenon of bound variable anaphora is a real phenomenon, appearing cross-linguistically in unrelated languages when the conditions allow it to be detected. We have also seen that its empirical distribution can be described by a number of high-level descriptive generalizations:

(29) The Scope Generalization: For a quantifier to bind a pronoun it must scope over that pronoun.
(30) The Command Generalization: A quantifier scopes over everything in the minimal finite clause it appears in.

Returning to the core issue, these generalizations appear to involve concepts that are quite specific to language: quantifier, binding, pronoun, scope, minimal finite clause. If we accept the generalizations in this form, it would seem that we are committed to highly domain specific analyses for this phenomenon. Indeed, that conclusion was adopted by generative grammar in some form in the 1980s and is consistent with a view of the evolution of language that sees it as an accretion of small evolutionary steps (e.g., Pinker and Bloom, 1990). However, current proposals derive these generalizations from more abstract principles and it is these, we believe, that should be evaluated for domain-specificity.

## 3. A theoretical account

Generative accounts of linguistic phenomena are couched at a level of analysis that is close to Marr's (1982) Computational Level. That is, the theory specifies a system that guarantees a particular pairing of sounds and meanings across a potentially unbounded domain. A helpful analogy would be an axiomatized theory for arithmetic, that can specify, for a potentially infinite set of pairs of integers, what the sum is. How people actually add, that is, how they use this system, is distinct from what the system is. The kinds of empirical effect described above, when structures are ambiguous or not between referential and bound variable interpretations of pronouns, is specified by the system at the computational level, rather than being a side effect of processing. How the system is put into use in parsing, production, etc., is a distinct question (Chomsky, 1967 et seq).

Within current generative grammar, one approach that has been taken to the core question of how to pair up particular linguistic forms of sentences with their meanings is the theory of Merge. Merge is a principle of structure generation that is incorporated into a theory of what legitimate syntactic structures can be. It says that a syntactic unit can be combined with another syntactic unit to make a new syntactic unit, providing unbounded resources for the use of language.

We can recursively define a syntactic unit as follows (cf. Chomsky, 1995):

(31)
  - Lexical items are syntactic units.
  - If A and B are syntactic units then Merge(A, B) = {A, B} is a syntactic unit.

This theory takes us from a finite list (of word-like atomic lexical items) to an unbounded set of hierarchical structures. (31) is a theory of what the legitimate structures in human language are, presumably neurally implemented (Embick and Poeppel, 2015). But these structures cannot be used as language unless they interface with the systems of sound and meaning. The definition of syntactic unit, incorporating Merge, in (31) is not sufficient for specifying language unless we add a set of principles for mapping those objects to interpretations in terms of sound and meaning. This is a point that often goes under-appreciated in literature, following Hauser et al. (2002), about whether language just consists of recursion.^2^

One such mapping principle has to do with the periodicity that regulates the transfer of syntactic object to the phonological and semantic systems: the idea is that this mapping takes place at certain points in the construction of a syntactic object (again, keeping to the computational level here). We will take these points to be finite clauses; though that is a simplification (Chomsky, 2008), it is sufficient for our purposes here. This is our first interface mapping principle:

(32) Transfer: Transfer the minimal structure containing the finite complementizer to phonological and semantic computations. Once a structure has been transfered, it is no longer accessible to further syntactic computation.^3^

The phonological and semantic computations transduce information delivered by the structure building system into forms that can be used by mechanisms of processing, production, planning, etc.

These two very general theoretical principles, Merge and Transfer, are motivated by empirical phenomena unconnected to bound variable anaphora. Merge is motivated by the need to capture basic constituency and hierarchy effects in human language, while Transfer (of finite clauses) is motivated by the special status finite clauses have in syntactic phenomena in general: they are the locus of subject case assignment, of semantic tense specification, and of locality domains for displacement operations (Adger, 2015, for review). However, these two ideas, as we will show, take us a long way in capturing the empirical distribution of the bound variable interpretation phenomenon, which we now turn to.

We notate syntactic units as sets. When a syntactic unit is transfered, the result is notated as a set, flanked by a phonological representation above and a semantic one below.

We simplify phonological representations massively by using orthographic representations and a simple concatenation operator ⌢ to represent string order. There is far more structure in phonological representations, including information about prosody, phonological phrasing, and segmental properties, but we will ignore this here.

We simplify semantic representations by using a simplified logical representation with variables and connectives augmented by a representation for natural language quantifiers. Following much work in semantics, as well as the discussion above, we take a quantified sentence to have three semantically contentful parts: a restriction, the quantifier itself, and a scope (Barwise and Cooper, 1981). These correspond to the computational operations described above: identifying a salient set in the context, quantifying over it, and determining whether a condition holds of the members of the set picked out by the quantifier. We notate these three parts, as is standard, by writing the quantifier plus the variable it binds, a colon, then the restrictor in square brackets followed by the scope in square brackets, thus:

(33) Q x:[…x…][…x…]

This set of simplifying assumptions about the interface mappings will suffice for our purposes here.

Now consider the derivation of the sentence in (34). This derivation should be understood as a computational specification of a sound-meaning pairing, much as a proof in logic is a computational specification of a theorem derivable from a set of axioms. This computational specification is part of a particular linguistic action [say an utterance of (34)], but does not causally determine the action.

(34) Noone said that he danced.
(35)
  - Merge(he, danced) = {he, danced}
  - Merge (that, {he, danced}) = Transfer, since *that* is a finite complementizer
                     that⌢he⌢danced ←PHON
                           {that, {he, danced}}
                             SEM→ y danced
    Here the hierarchy partly determines order and the pronoun is semantically translated as the variable y.
  - Merge(said, {that, {he, danced}}) = {said, {that, {he, danced}}}
  - Merge(Noone, {said, {that, {he, danced}}}) =
          noone⌢said⌢that⌢he⌢danced ←PHON
                {noone, {said, {that, {he, danced}}}}
      SEM → No x:[x is a person][x said y danced and
                                         x=y]

As the phonological and semantic information is transfered to the relevant interfaces, information about linear order, pronunciation, and semantic interpretation accretes. Crucially, the statement that the variable x has the same value as y is added within the scope of the interpretation of the quantifier *noone*, just as in the informal paraphrase given in the last section. This ensures that it is interpreted as bound. Of course, we can equate x to another variable not in the scope of the quantifier, in which case we get the referential reading, thus accounting for the core ambiguity we began with. Equation of variables in itself could conceivably be a purely semantic, possibly non-linguistic process, at the heart of anaphoric dependency of all sorts, but the bound interpretation is constrained by how the building up of structures interacts with their interpretation.

Now let us look at a case where variable binding is not possible:

(36) Friends that no woman knew said that she danced.

In the following derivation, steps (a–c) build up the verb phrase *said that she danced* and steps (d–h) independently build up the subject *Friends that no woman knew*. Although (d–h) is ordered after (a–b), this is just an artifact of writing down the derivation. One can think of these as separate derivations taking place in parallel.

(37)
  - Merge(she, danced) = {she, danced}
  - Merge (that, {she, danced}) =
                     that⌢she⌢danced ←PHON
                           {that, {she, danced}}
                              SEM → y danced
    Steps (a-b) build up the embedded clause *that she danced*, which contains the pronoun of interest.
  - Merge(said, {that, {she, danced}}) = {said, {that, {she, danced}}}
  - Merge(knew, friends) = {knew, friends}
  - Merge(no, woman) = {no, woman}
  - Merge({no, woman}, {knew, friends}) = {{no, woman}, {knew, friends}} This part of the derivation builds up the relative clause *that no woman knew*. Note that the item *friends* is Merged with the verb *knew*, which is why it is interpreted as the object of that verb. However, the actual relative clause has a gap in the object position. This necessitates the next part of the derivation:
  - Merge(that, {{no, woman}, {knew, friends}}) = {that, {{no, woman}, {knew, friends}}}
  - Merge(friends, {that, {{no, woman}, {knew, friends}}}) =
    The subject *friends that no woman knew* involves a further Merge operation that takes the object of the verb *knew*, which is the unit *friends*, and Merges it with the whole structure *that no woman knew friends*. This happens in English because of a property of relative complementizers that triggers this displacement. Languages vary in whether relative clauses involve this kind of displacement Merge, with some leaving the object in its base position (Cole, 1987).
    At this point, the whole relative clause is built up. Following the Transfer principle, what is transfered is the unit containing the relative complementizer *that*:
               that⌢no⌢woman⌢knew ←PHON
       {friends, {that, {{no, woman}, {knew, friends}}}}
         SEM → λy: No x:[x is a woman] [x knows y]
    In English, as just mentioned, only the higher of the two occurrences of *friends* is pronounced. In other languages, the lower occurrence is pronounced. We do not know of languages where both occurrences are pronounced. This suggests another mapping principle:
    **Pronounce Once**: When a single object appears at more than one position in a structure, pronounce only one instance.
    This principle, together with the Transfer principle, gives us the phonological representation above.
    The semantics associated with this piece of structure is the tripartite structure we are familiar with, whose domain is restricted to a set of women, and whose scope is the verb phrase of the relative clause (basically the verb *knew* and its object). We adopt a standard approach to relative clause semantics (Heim and Kratzer, 1998): the transfered object *friends* is just translated to a variable bound by the relative complementizer *that*, and we notate this semantics in the standard way as λy:[…y…].
  - Merge({friends, {that, {{no, woman}, {knew, friends}}}}, {said, {that, {she, danced}}})
    friends⌢that⌢no⌢woman⌢knew⌢said⌢that⌢
                      she⌢danced ←PHON
    { { friends, {that,{ {no, woman}, {knew, friends}}}},
           {said, {that, {she, danced}}}}
        SEM → some y: [y are friends and No x: [x is a
             woman] [x knows y]] [y said w danced]
    The final chunk of the derivation combines the whole subject with its VP. The VP is built up in step (c), and the output of that is Merged with the output of step (h). Phonologically, we simply concatenate these in the order required by English. Semantically, we take the bare noun *friends* to be interpreted with an existential quantifier *some*. We identify the variable this quantifier binds with that of the relative clause, and that is the variable that is the subject of the verb phrase. The pronoun in the embedded clause is translated as a further variable.

At this point, however, it is not possible to connect x and w, since the interpretation of the quantifier phrase *no woman* has already been completed, and the variable x has been fully interpreted, before w is encountered. This derives the simple cases of the Scope Generalization directly from very general principles of the relationship between syntax and semantics: the pronoun cannot be interpreted as bound unless it is computed within the scope of the quantifier.

The more outré effects of the Scope Principle are also amenable to the same set of basic principles. Recall that a quantifier can scope over everything inside the finite clause it is immediately contained within. With this in mind, consider the derivation of (38):

(38) She believed that every author danced.
(39)
  - Merge(every, author) = {every, author}
  - Merge({every, author}, danced) = {{every, author}, danced}
  - Merge(that, {{every, author}, danced}) = {that, {{every, author}, danced}}
            that⌢every⌢author⌢danced ←PHON
                  {that, {{every, author}, danced}}
          SEM → Every x:[x is an author][x danced]
  - Merge(believed, {that, {{every, author}, danced}}) = {believed, {that, {{every, author}, danced}}}
  - Merge(she, {believed, {that, {{every, author}, danced}}}) = {she, {believed, {that, {{every, author}, danced}}}}
        she⌢believed⌢that⌢every⌢author⌢danced
                                  ←PHON
    {she, {believed, {that, {{every, author}, danced}}}}
    SEM → y believed that Every x:[x is an author][x
                                    danced]

The variable x is fully computed with values assigned, before y is introduced. It follows that the meaning of the pronoun *she* cannot depend on the quantifier, so the bound variable interpretation is correctly predicted to be unavailable.

Compare this to the following case:

(40) Every author's publicist loved her.
(41)
  - Merge(every, author) = {every, author}
  - Merge({every, author}, publicist) = {{every, author}, publicist}
  - Merge(loved, her) = {loved, her}
  - Merge({{every, author}, publicist}, {loved, her}) = {{{every, author}, publicist}, {loved, her}}
  - Merge({every, author}, {{{every, author}, publicist}, {loved, her}}) =
      every⌢authors⌢publicist⌢loved⌢her←PHON
         {{every, author}, {{{every, author}, publicist},
                                 {loved, her}}}
          SEM → Every x:[x is an author][THE y:[y is
                 publicist of x][y loves w and w=x]]

In step (e), the Merge operation allows the quantifier phrase *every author* to scope, in its finite clause, higher than the pronoun. This computational step is usually called Quantifier Raising, and is a syntactic way of marking the semantic scope of the quantifier, but in the theoretical system it is just another application of the operation Merge.

Just as we saw with the relative clause case, a single syntactic unit (in this case the quantifier phrase *every author*) is Merged with the larger unit that contains it, creating two occurrences of the phrase. One occurrence of this quantifier phrase is now high in the structure. This means that when its semantics is computed, it takes scope over the whole clause. The upshot of this is that the variable introduced by the pronoun is introduced at a point where the variable bound by the quantifier is still being computed. This allows them to be identified (notated here as w = x) and the bound variable reading to arise.

On the phonological side of the computation, one of the occurrences of the quantifier phrase is not transfered to the phonological component following the mapping principle Pronounce Once (just as we saw with the relative clause). For the case of quantifiers in English, it is the higher rather than the lower occurrence that is not transfered, giving us the effect that the quantifier is interpreted high in the structure, but pronounced low. No extension of the computational technology already appealed to is necessary to capture this. Which occurrence is pronounced is a point of cross-linguistic variation; for example in Hungarian the higher occurrence is pronounced (see Kiss, 1981).

We might ask whether we could follow the same kind of derivation we have just seen, and allow the quantifier to Merge higher in (38), hence generating the unattested binding possibilities. However, recall that transfer applies to finite clauses and that once a finite clause is transfered, no further computation is possible. Given this, the quantifier phrase in (38) cannot be moved to a position where it scopes over the pronoun.

The principles sketched here are sufficient to capture the phenomena we have surveyed. The effects of the Scope and Command Generalizations emerge from possible Merge operations interacting with the way that finite clauses are transfered to the phonology and the semantics. We have suceeded in making the descriptive generalizations special cases of much more general principles of structure building and how structures are mapped to the interfaces. We have not shown here how these more general principles play a role in explanations of other phenomena, as this would entail a book rather than a paper. However, these general principles of structure building and mapping to the interface are effective in deriving a slew of generalizations about the syntactic structure of human languages.

### 3.1. Further predictions

The theoretical work we have just done, however, goes beyond our core generalizations, because bound variable interpretations interact in a complex way with other phenomena. The following cases do not follow from the generalizations directly, but they do follow from the theoretical system:

(42)
  - Which of his relatives did the sybils decree that no man may love?
  - Which of his relatives forced the sybils to decree that no man was innocent?

In (42-a), the pronoun can receive a bound variable interpretation, which is not available in (42-b). Why should this be?

Consider (42-a) in more detail. It includes the phrase *which of his relatives*, which is interpreted as the object of the verb *love* in the embedded clause. This entails that it is initially Merged with *love* in a derivation that then later involves the Merge of *no man*. The phrase *which of his relatives* is then Merged again with the finite clause, and the remainder of that finite clause is transfered to the phonological and semantic systems, just as we saw for relative clauses above. This means that our derivation will reach a point that looks as follows (we do not show the internal structure of *which of his relatives*):

(43)
                        that⌢no⌢man⌢love⌢← PHON
  {{[which of his relatives]}, {that, {{no, man}, { may {love, {[which
                                    of his relatives]}}}}}}
  SEM → y: no x:[x is a man][x may love y: y is a relative of z and
                                               z=x]

Here the variable z is introduced for the pronoun *his* at a point in the computation where the phrase *which of his relatives* is in the scope of the quantifier phrase *no man*. When the finite clause *that no man may love* is transfered, the syntactic unit *which of his relatives* is in the object position, and so what is transfered to the semantic computation is a structure where the pronoun's interpretation is computed within the computation of the quantifier phrase. Because of this, we can add the condition that z = x, where x is the variable introduced by the quantifier phrase. The higher occurrence of the phrase *which of his relatives* then undergoes further Merge, after the introduction of the material in the higher clause, to derive the whole sentence with the bound reading.

Compare this, however, to (42-b). Here the phrase *which of his relatives* is the subject of the higher verb *force*. It is never, therefore, in the scope of the quantifier phrase *no man* at any point in the derivation, and there is therefore no means of allowing the pronoun *his* to be bound by that quantifier. The underlying system of computations that build structure and transfer it to phonological and semantic systems correctly predicts a rather sophisticated distribution of form-meaning relations, going well beyond the basic descriptive generalizations.

We have now come most of the way through the argument. We have introduced the phenomenon of bound variable readings and seen that it is present cross-linguistically; we have outlined the core aspects of the phenomenon and shown how the descriptive generalizations about the phenomenon derive from a theoretical account built on deep, abstract principles stated at a computational level of analysis that specifies the sound-meaning relationships for an unbounded set of structures. We have also shown how that system extends to the interactions between bound variable anaphora and other syntactic and semantic phenomena. Before we evaluate the domain-specificity or domain-generality of these principles, however, we should ask whether there is a compelling alternative account of this phenomenon that does not appeal to operations that build and interpret structure.

### 3.2. A cognitive grammar account

The answer to this question is that there is not. The only in depth discussion of the phenomenon that is non-generative and covers a similar range of empirical phenomena is van Hoek (1996), who provides an investigation of bound variable anaphora within the framework of Cognitive Grammar. Van Hoek argues that whether a pronoun can be bound is dependent on the salience or prominence of the quantificational antecedent. For the relevant cases, she defines salient as occupying the Figure in a Figure-Ground structure. Figure Ground relations are plausibly used across cognition (Talmy, 1975). The Figure Ground relationship is conceived of purely semantically in van Hoek's work. We give here a standard specification of how this relation is to be understood within language (Talmy, 2000, p. 312):

(44)
  - The Figure is a moving or conceptually movable entity whose path, site, or orientation is conceived as a variable, the particular value of which is the relevant issue.
  - The Ground is a reference entity, one that has a stationary setting relative to a reference frame, with respect to which the Figure's path, site, or orientation is characterized.

No doubt the notion of Figure-Ground relation is an important semantic schema in cognition. However, contrary to van Hoek's proposal, it does not seem to be implicated in defining salience for bound variable anaphora. There are numerous cases where the subject of a sentence is the Ground, rather than the Figure but this does not impact on the distribution of bound variable anaphora.

Talmy gives examples such as *the room filled with smoke*, where the Figure is the smoke which moves or changes with respect to the room, which is therefore the Ground. In van Hoek's approach, we would expect the object to act as a salient antecedent for a pronoun in the subject position, but this is not what we find, using examples modeled on Talmy's pattern *Ground filled with Figure*:

(45)
  - Each room filled with the scent of the flowers in its center.
  ^*^Its vase filled with each blooming flower.

Here we find that a quantifier phrase which is semantically the Ground can bind a pronoun in the Figure, and conversely that a quantifier phrase that is the Figure cannot bind a Ground pronoun.

The verb *contain*, by definition, also has a Figure as object and Ground as subject. Again, if the Figure is always salient, van Hoek's system incorrectly predicts the wrong binding possibilities:

(46)
  - Each book contains its author's biography as an initial chapter.
  ^*^Its initial chapter contains a synopsis of each book.

Some action verbs, especially those of consumption, have been analyzed as involving a Figure object moving with respect to a Ground subject. Once again, the binding patterns we see empirically are unexpected on an approach like van Hoek's.

(47)
  - Each giant gobbled up his own child.
  ^*^His child gobbled up each father.

In all of these cases, the Figure is the object, and hence, in van Hoek's proposals, the possible binding relations should have exactly the reverse distribution from the standard cases. One might try to rescue the system by proposing some special semantic relation to be associated with subjecthood that overrides Figure-Ground relations, but that, of course, would be circular in the absence of an independently verifiable, purely semantic specification for what a subject is. Van Hoek provides no such specification.

One might attempt to supplement van Hoek's proposal by appealing to information structure effects on salience. For example, we could ensure that the relevant set of books is pre-established in the context, and that universal quantification over this set is also pre-established, and further we can ensure that the quantifier phrase is a Figure. But still the structural facts override all of these potential cues and are determinant of what the binding possibilities are. Binding from a highly salient Ground object into a pronoun in the subject position is impossible:

(48) There are a whole lot of new books on display at the convention this year and they've all got something in common: ^*^Its initial chapter contains a synopsis of each book.

We do not want to deny that pragmatic principles may have an impact on the processing of bound variable anaphora as it is clear that this is a factor in understanding the full empirical range of effects (Ariel, 1990). Effects of temporal order (the quantificational binder normally precedes the bound pronoun, though see (42-a) for an example of the opposite) may well fall into this category. However, such principles do not, by themselves, explain the empirical distribution of the phenomenon.

There is a larger issue connected to domain specificity that emerges from attempts, like van Hoek's, to explain bound variable anaphora, and other syntactic phenomena, by appeal to non-structural, cognition-wide, properties. Structure, when at play, always trumps the effect of semantic, informational, pragmatic or social properties. If phenomena are not structurally constrained, then we need explanations for why such factors do not regularly play a part in determining bound variable anaphora.

Languages vary according to what kinds of expressions can be bound by quantifiers (Déchaine and Wiltschko, 2014), but they are always restricted structurally. This is striking, especially since pronouns can refer to entities which are salient or prominent in the discourse context in a variety of ways.

For example, pronominal elements like *that* and *it* can be differentiated by a measure of givenness (Gundel et al., 1993). According to Gundel et al. (1993), *it* refers to the focus of attention in the discourse at the time, whereas *that* picks out a referent which is “activated” in the discourse, i.e., brought into current short-term memory, normally by being mentioned, but is not in the focus of attention. This is illustrated in the following pair (modeled on examples from Gundel et al.), where the subject in (49-a) is naturally understood as the focus of attention and can be referred back to by *it*. In contrast, the dog in (49-b) is not naturally understood as the focus of attention, and hence *it* is infelicitous in the continuation (as indicated by #), but since the referent is activated, it can be referred to by *that*.

(49)
  - My neighbor's rottweiler chased my cat this morning. It's the same dog that ate my cat's food last week.
  - Ikea delivered playground equipment to my neighbor with the rottweiler this morning. #It's the same dog that ate my cat's food last week [ok: That's the same dog that ate my cat's food last week].

Since notions like focus of attention are linguistically relevant in the choice of *it* vs. *that*, we might expect to find a language in which the same categories of givenness are relevant to quantifier binding. For example, the focus of attention, if quantified, would be able to bind a pronoun, as in the following example.

(50) Every one of my neighbor's dogs chased one of my cats. #It's the same dog that ate the cat's food last week.

Here, the bound reading would be that there are pairings of dogs and cats, where the dog that chased a cat also ate that particular cat's food. Such a reading is impossible in English as seen in (50), even though the quantified subject is in focus and should therefore be a legitimate antecedent for English *it*.

Compare the salient bound reading when the structural conditions on quantifier binding are met, in (51).

(51) Every dog chased one of my cats before it ate the cat's food.

What is important is not that English doesn't allow a bound reading in (50), it's that no language has been reported which does. This suggests that the mechanism for assigning reference to pronouns is not highly variable, across languages; it can pick up a non-quantified focus of attention without structural conditions, as in (49-a), but it can be bound by a quantifier only when introduced in the phase of the derivation in which the quantifier is interpreted, as argued above and as illustrated by the infelicity of (50).

In fact, much more exotic language systems are imaginable, and it is quite striking that they are unattested. There is remarkable cultural diversity, for example concerning how important social hierarchy is to a society. Some societies have complex systems of rank and class and their languages have complex ways of encoding respect and deference and entitlement, as in Japanese. Other societies are relatively egalitarian and their languages lack these honorifics, for example traditional Khoi society (Lee, 1979).

If languages interacted with general cognition in unrestrained ways, we might expect to find a language in which the honorific system was so important that it mattered for aspects of syntax such as quantifier binding. Imagine a language in which only socially superior entities could bind quantifiers. In this language, a speaker could have a bound reading for (52-a), but (52-b) could only have the referential reading for the pronoun.

(52)
  - Every nobleman called to his slave.
  - Every slave called to his master.

Once again, such a language is unattested, suggesting that at least some aspects of pronominal reference resolution are language specific, and not permeable to arbitrary cognitive domains.

## 4. Domain specific, domain specialized or domain general?

The purpose of this section is not to argue that the principles (Merge, Transfer, Pronounce Once) so far discussed are, or are not, specific to language, but rather to sketch out the kinds of issues that can be addressed, and directions for investigation that can be pursued, once principles with explanatory depth and empirical reach are established. The principles we have identified can easily be understood as specific to language (a traditional view). This section argues that it is perhaps possible to understand them as language-specialized versions of very general cognitive and computational factors, though this is speculative. Crucially, however, these principles are mysterious when viewed from the perspective of communication, interaction, and general learning, concepts which provide little theoretical traction on important empirical phenomena of syntax and semantics.

Explanation of the unbounded link between structure and meaning requires a recursively specified procedure, or its equivalent. This is an underappreciated point. Some cognitive mechanism must be able to generate, and not simply retrieve, a form-meaning pair, since the number of such pairs is both in practice and in principle too large to store. Once there is such a mechanism, there is a generative system that restricts the possible form-meaning pairings. The fact that some structures (for example those involving center embedding of elements of the same category) are difficult to process, or are never used, is irrelevant to the question of whether there is such a procedure, for reasons understood since Miller (1956), contra Christiansen and Chater (2015). The particular formation of Merge we have given, in addition, generates constituent structures with maximal levels of branchingness (two, for the formulation we adopt here) and a scaffolding on which to hook compositional construction of meaning. We have modeled this operation as a set formation operation applying to elements in a restricted domain. We will also use the term Merge as the name for the modeled physical properties.

As we have presented Merge, its domain is restricted to what we called “syntactic units” in (31). It operates on discrete linguistic units (morphemes or words) to create larger, structured, discrete units (phrases). There seem to be few other cases of systems displaying this kind of generative nature elsewhere in human cognition. Arithmetic and tonal music have been discussed as recursive generative systems that involve similar structure-building operations (Hurford, 1987; Rohrmeier, 2008). Suppose that we posit A-Merge and T-Merge alongside L-Merge for the structure-building operations involved in arithmetic, tonal music, and language, respectively. The difference, if any, would lie in what domain these various kinds of Merge are restricted to: tonal music combines elements with sound but no meaningful content, and arithmetic combines elements with abstract content (which can be counted) but no fixed sound, while language combines elements which are pairings of meaning and sound (or other externalizable form).

Humans have natural capacities for arithmetic and tonal music differing substantially from the natural abilities of the other primates (Tomonaga and Matsuzawa, 2000; Carey, 2001). There is good evidence, in fact, that nonhuman primates lack Merge (Yang, 2013), which entails that there was an evolutionary event which led to human brains having Merge. At the same time, many cultures do not develop any arithmetic (Izard et al., 2008) or tonal music (Lomax, 1968; Wallin et al., 2000), so it is fairly clear that the pressures of natural selection could not have led to humans as a species having these particular abilities (as Darwin, 1871, noted). One is led to the conclusion that either A-Merge and T-Merge are the same thing as L-Merge, or biproducts of it, or else a single evolutionary event led to all of the different kinds of Merge. These three apparent alternatives may simply reduce to a matter of how the terms are defined.

It is clear that language is used as a communication system and that it makes central use of Merge; but it is less clear that Merge-based communication provided an evolutionary advantage that caused Merge-endowed brains to be selected for. It is just as plausible that Merge-endowed brains had some other advantage, for example in planning, or in reasoning, or in memory. In fact, Chomsky (1966, 2010) has speculated that the generative system of language might essentially be a system of thought, not of communication; communication would be something one can do with language, once it is “externalized” (i.e., pronounced audibly, or articulated visually or tactilely).

This scenario changes the terms of the question of whether Merge is specific to language; language in fact takes on a much larger role as a central part of cognition. In this scenario, Merge is not specific to language-qua-communication system. Merge is rather a property of a more general system of symbolic thought, a core component of language understood to be a generative system. The question of whether arithmetic and/or tonal music are also instantiations of it is secondary, since plausible evolutionary paths suggest that arithmetic and tonal music were not causally central to the philogenetic emergence of Merge.^4^

We might consider the other two principles we appealed to in an analogous manner. These principles govern the way that the structures generated by Merge are interpreted by phonological and semantic systems. The first of these principles is the following.

(53) Transfer: Transfer the minimal structure containing the finite complementiser to phonological and semantic computations. Once a structure has been transfered, it is no longer accessible to further syntactic computation.

This principle actually has three components: (i) it imposes a periodicity on the transfer of information between the structure creating and the interpretive systems; (ii) it imposes an opacity condition so that transfered structure is inaccessible for further computation; (iii) it specifies finiteness as a flag for the application of transfer. We take these in turn.

The theoretical architecture we defended as an analysis of bound variable anaphora (and many other syntactic phenomena) is stated, as we said, at the computational level—it specifies what function is computed. But the particular principles we have used are fundamentally computational in a different sense too: they involve the alteration of discrete structures according to a set of rules applying to these structures. Merge creates and manipulates an unbounded set of discrete structures of certain forms from a finite list of discrete inputs (roughly, abstract representations of words or morphemes). It is the computational nature of Merge that allows it to provide an explanation for the fundamental fact that human languages can be unbounded in how they connect forms to meanings. Periodicity in computation, a core aspect of (53), is plausibly a general natural law, going beyond domain general laws of cognition (Strogatz and Stewart, 1993). Periodicity also appears to be ubiquitous in biological phenomena, possibly evolving as a side effect of efficiency conditions relating successful organisms to their environments within constraints imposed by physical law (Glass and Mackey, 1988). It is certainly speculative, but at least the periodicity part of (53) may be a factor that is domain general, not only with respect to human cognition, but also to physical or computational systems in general. If that is true, then the organization of information transfer between Merge-built structures and other systems of the mind is not language specific, not cognition specific, not human specific and possibly not even biology specific.

However, there is more to (53) than just the periodicity of the transfer of syntactic information. There is also the notion that syntactic information, once transfered, is no longer accessible to further computation. This idea is not only important in capturing the limitations on quantifier scope, but also for locality effects elsewhere in syntax, such as the ubiquitous locality effects seen in long-distance dependencies (Chomsky, 1973, et seq). Locality of this sort may also be reducible to more general properties. Any computational system requires organized space (such as a look-up table), which stores information that is used multiple times in a computation. Again, there is some speculation here, but it does not seem implausible that such storage space is limited in human cognition, so that once the syntactic information is transfered, the relevant storage space is no longer available at the next stage of the computation. Working memory in other areas of human cognition (when used, for example, in processing language or other information) is known to be restricted (Miller, 1956; Baddeley, 1992); storage space in the computation that defines well formed structures in a language may be likewise restricted. This would be a case of a general principle of space optimization, which applies across cognition and hence is domain general, operating in a specialized way within the syntactic system to restrict the space available for computation.

It is important, however, to note that these domain general principles (periodicity and space optimization) are applying to linguistic data structures (structures generated by Merge) not as principles of processing, but at a Marrian computational level, as principles that constrain the range of possible syntactic objects. We draw much the same lesson here as we did in our discussion of Merge: the same abstract principle may be at work in different domains of cognition, and how it plays out in those domains will be affected by the nature of the primitives of those domains. So the operation of the principles is specialized to the particular structures in the relevant domain, but the principles themselves may be entirely general.

The final aspect of the Transfer principle we have not discussed is the idea that it involves finiteness. Finiteness appears to be a formal property, with some connection to both meaning (especially to the interpretation of tense) and to morphological form (the shape of complementizers, case assignment etc.), but it operates within the syntactic system independently of them (see Adger, 2007, for linguistic evidence). Further linguistic investigation is required to understand the relationship between quantifier scope and finiteness, especially since not all languages mark finiteness overtly, but all languages seem to restrict the scope of quantifiers in similar ways. We think it likely that there will be some formal specification of the point of transfer, as empirically quantifier scope seems to always respect finite clause boundaries when they are detectable, but exactly how this plays out across a richer range of languages is still something of an open question.

The final principle we appealed to in our explanation of the workings of bound variable anaphora is the following.

(54) Pronounce Once: When a single object appears at more than one position in a structure, pronounce only one instance.

This principle is at work in the interpretation of structures where a single syntactic unit is present at two distinct places in the generated structure. Phenomenologically, we hear a single pronunciation of some constituent, but there is linguistic (including psycholinguistic) evidence for its presence elsewhere in the structure. We saw this principle at work in our analysis of (55) [repeated from (42-a)]:

(55) Which of his relatives did the sybils decree that no man may love?

The bound pronoun *his* behaves as though it is in the scope of *no man*, although the phrase it is embedded within (*which of his relatives*) is clearly not in a surface position that would allow that. The solution is to take *which of his relatives* to be Merged with *love*, where *no man* can scope over it, and then to Merge again, ending up in its surface position. Independently, we also need to explain how this phrase is interpreted as the object of the verb *love*, so the proposal that it Merges with that verb is motivated. There is good psycholinguistic evidence that the human sentence processing mechanism is sensitive to the presence of a single constituent in multiple positions in the parse-tree it constructs while comprehending a sentence with such long-distance dependencies (Lewis and Phillips, 2015, for review and references) and that it detects unpronounced constituents in general (Cai et al., 2015), providing evidence from processing for this linguistic analysis.

(54) is stipulated here as a language specific principle. It applies to realize syntactic objects as phonological objects in a way that is dependent on the nature of the structure. Chomsky (2013) has speculated that it might be understood as emerging from a particular kind of reduction of computation, perhaps minimization of the phonological computation that is required. On the assumption that a series of phonological rules need to apply to the output of the syntax, if there are two instantiations of the structure, the same phonological rules will have to apply to both instantiations, increasing the amount of computation. If there are a great many dependencies to be formed in a particular structure, the same phonology would appear multiple times. If the phonological computation can simply be done once, phonological processing is dramatically reduced.

It seems unlikely, as Chomsky notes, that this principle is functionally motivated to enhance parsing, as the absence of a phonological signal marking a grammatical dependency like a relative clause, is inimical to constructing the correct parse. Similarly, this principle applied to quantifier scope leads to an increase in grammatical ambiguity, again a property which would seem difficult to motivate on functional grounds.

If this principle is not functionally motivated by communicative or parsing pressures, might it be exapted from elsewhere in cognition, as we suggested for aspects of periodicity and locality? It is certainly the case that a fundamental aspect of human cognition is the keeping track of an identical object in time and space. Leslie et al. (1998) propose an internal representation for objects that functions as an index (much like pointing) and use this to explain the relationship between perceptual and conceptual representations of objects (cf. Pylyshyn, 1989). Speculating again, it may be the case that a mechanism that is used for objecthood in a domain outside of language is at play, though the structures to which it applies are linguistic, rather than visual or conceptual. If this is the case, then the index is phonologically realized, but points to different instances in syntactic space of the same syntactic unit. Once again, a cognition general property is specialized to the way that linguistic knowledge is structured.

The suggestions we have made are speculative, but the core point is that by developing theoretically deep explanations of linguistic phenomena, we can begin to evaluate the domain specificity of the abstract principles proposed in the knowledge that these principles are solidly based in the empirical phenomena of language.

## 5. Conclusion

We have outlined a general phenomenon at the syntax-semantic interface, shown how it is cross-linguistically valid, provided both a descriptive outline of its empirical properties and a theory of some depth explaining why those properties are as they are. We have also argued that no reasonable alternative (currently) exists. All current approaches that achieve a good level of empirical success are generative in a sense recognizable from the kind of theory we sketch here (although they may be expressed in different generative frameworks, such as Categorial Grammar, Jacobson, 1999, or Lexical Functional Grammar, Dalrymple et al., 1997).

This paper makes a methodological point and a theoretical point. The methodological point is that principles to be evaluated for domain specificity should be principles that actually do explanatory work in capturing linguistic phenomena. That is, we need to understand the nature of the linguistic phenomena first, and use that understanding to ask more general questions of cognitive science. Any alternative approach that ignores or dismisses a vast range of empirically impeccable work, and attempts to show that some proposed principle of communication or learning explains something general about language is insufficient. Any such alternative needs to have, or at very least be in principle capable of extending to, the kind of empirical coverage and explanatory depth of current generative linguistic theory.

The more theoretical point we have made is that three core principles, motivated from work in theoretical linguistics, when evaluated in terms of domain-specificity suggest something interesting. At a very abstract level, some of these principles may well be at play outside of the human language faculty, as principles of the optimization of space, periodicity of information transfer, and object identity. However, when instantiated in the human language faculty, they operate over linguistic entities created by Merge. Merge itself, we argued on the basis of cross-species comparison, appears to be unique to humans and therefore the result of some evolutionary event. It is not obvious that Merge plays a role elsewhere in human cognition (aside, perhaps, in possibly language-related areas such as music and arithmetic), or in natural law more generally, but further investigation may change our current perspective on this.

What does this discussion have to contribute to the question of whether there is an innate, language specific cognitive system? It suggests that there are principles that play a role in explaining empirical linguistic facts which may be language-specialized versions of more general cognitive principles. The human brain, then, appears to be set up in a way that involves the canalized development of such specialization. That is part of, if not the whole of, Universal Grammar.

## Funding

Arts and Humanities Research Council of the United Kingdom (Grant AH/G109274/1: Atomic Linguistic Elements of Phi).

### Conflict of interest statement

The authors declare that the research was conducted in the absence of any commercial or financial relationships that could be construed as a potential conflict of interest.

## Footnotes

^1^There is one final aspect to the phenomenon of bound variable interpretations which is not captured by scope and command: sometimes a quantifier phrase can take scope over a pronoun, but it cannot bind it. If all that is required is the Scope Generalization, examples like the following should be well formed with a bound variable reading:

(i)
  - She loved every author.
  - He killed each man.
(ii)
  - Her publicist loves every author.
  - His friend killed each man.

The examples in (i) do not have bound variable readings. This doesn't follow from what we have said so far. These phenomena (noted for questions by Postal, 1971, extended to quantifiers by Chomsky, 1976, and dubbed Strong and Weak Crossover, respectively) cannot be captured by the Scope and Command Generalizations alone. Various approaches have been taken to this phenomenon, the Weak Crossover case is variable across languages, and there is no clear consensus on its analysis. We will not attempt to capture this data here.

^2^In their words, “We propose in this hypothesis that FLN [the faculty of language in the narrow sense] comprises only the core computational mechanisms of recursion as they appear in narrow syntax *and the mappings to the interfaces*” (Hauser et al., 2002, p. 1573) [emphasis ours].

^3^What constitutes a finite complementizer across languages will be left unspecified here. For English, the embedding finite complementizer is *that*, while matrix finite clauses have no pronounced finite complementizer. In the derivations below, we'll simply assume that finite matrix clauses are transfered once completed, but it should be borne in mind that technically there is a more complex syntax involved. There is also a question to be answered about relative clauses, where, as we will see the minimal structure containing the finite complementizer is transfered only once the particular requirements of that complementizer are all satisfied. This provides certain elements (in wh-questions, topicalizations and relative clauses, but not quantifiers) with a limited capacity to evade locality effects. We will abstract away from these further details here.

^4^Though there are proposals that accord musical ability a more central role in language evolution, cf. Darwin (1871), Brandt et al. (2012) and references there.
